# Left ventricular systolic function in Nigerian children infected with HIV/AIDS: a cross-sectional study

**DOI:** 10.5830/CVJA-2015-066

**Published:** 2016

**Authors:** Ijeoma Arodiwe, Ikefuna Anthony, Obidike Egbuna, Ibeziako Ngozi, Ejikeme Arodiwe, Bennedict Anisuba, Sunday Omokoidion, Christy Okoroma

**Affiliations:** Department of Paediatrics, University of Nigeria Teaching Hospital, Ituku-Ozalla, Enugu, Nigeria; Department of Paediatrics, University of Nigeria Teaching Hospital, Ituku-Ozalla, Enugu, Nigeria; Department of Paediatrics, University of Nigeria Teaching Hospital, Ituku-Ozalla, Enugu, Nigeria; Department of Paediatrics, University of Nigeria Teaching Hospital, Ituku-Ozalla, Enugu, Nigeria; Department of Medicine, College of Medicine, University of Nigeria, Enugu, Nigeria; Department of Medicine, College of Medicine, University of Nigeria, Enugu, Nigeria; Department of Paediatrics, University College Hospital, Ibadan, Nigeria; Department of Paediatrics, College of Medicine, University of Lagos, Lagos, Nigeria

**Keywords:** left ventricular systolic function, HIV/AIDS, children, echocardiography, Nigeria

## Abstract

**Background:**

Cardiac complications contribute significantly to morbidity and mortality in children with HIV/AIDS. These rates have been under-reported in sub-Saharan African children.

**Methods:**

This was an observational, cross-sectional Doppler echocardiographic study of ventricular systolic function, performed at a tertiary clinic on children with HIV/AIDS.

**Results:**

Left ventricular systolic dysfunction was present in 27.0% of the children with HIV infection and 81.2% of those with AIDS. The mean fractional shortening in the AIDS group (31.6 ± 9.5%) was significantly lower than in the HIV-infected group (35.3 ± 10.5%, *p* = 0.001). A significant correlation was found with CD4+ cell count and age, and these were the best predictors of left ventricular systolic dysfunction in the stepwise multiple regression analysis (*r* = 0.396, *p* = 0.038; *r* = –0.212, *p* = 0.025, respectively).

**Conclusion:**

Left ventricular systolic dysfunction is common in Nigerian children with HIV/AIDS.

## Background

Human immune deficiency virus (HIV) infection and its effect, acquired immune deficiency syndrome (AIDS), is one of the most frightening emerging diseases and constitutes a global health burden with overwhelming social, economic and political repercussions.^1^ It is one of the challenges facing African countries today, as most countries in sub-Saharan Africa have generalised epidemics, defined as prevalence rate > 1%. It is a leading cause of death and shortened life expectancy in this region.^2^

This disease is characterised by a deficient cell-mediated immunity.^3^ The manifestation is usually protean, as shown by varied clinical features seen in different parts of the world.^4^ It results in a progressive dysfunction of multiple organ systems.^5^ In sub-Saharan Africa where the burden of the disease is very high, involvement of the heart in HIV has become a clinical problem over the last decade, but there are few published studies on it, especially in children.^6^-^8^

Left ventricular dysfunction is important in the clinical history and prognosis of HIV infection.^9^ It is most often clinically silent in HIV/AIDS patients and can progress to symptomatic left heart failure.^10^ Median survival to AIDS-related death is 101 days in patients with left ventricular dysfunction, and 472 days in patients with a normal heart, as shown by echocardiography at a similar infection rate.^11^ Reduced left ventricular fractional shortening and increased wall thickness were also predictive of survival after multivariate adjustment.^11^ With improved clinical surveillance and treatment, using highly active antiretroviral therapy (HAART), more patients are surviving potentially fatal opportunistic infections, only to succumb to neoplasm or end-organ damage. Heart muscle disease is one such end-organ damage.^12^

Our study evaluated left ventricular systolic function (LVSF) and factors affecting it in children with HIV and AIDS, compared with age- and gender-matched HIV-negative controls, using M-mode, two-dimensional and Doppler echocardiography.

## Methods

This was a descriptive, cross-sectional study of 90 paediatric HIV and AIDS patients, aged between 18 months and 14 years. Their age and gender matched the HIV-free controls. The cases were seen at the University of Nigeria Teaching Hospital (UNTH), Enugu, from February to December 2011. The study was carried out at the Paediatric retroviral clinic and in the paediatric wards. Those in the wards are already confirmed to be HIV positive or have AIDS. The controls were recruited from the children’s out-patient department, immunisation and adolescent clinic.

The patients had a pre-echocardiography evaluation to identify those qualifying for the study. The inclusion criteria were children who were HIV 1 and/or 2 positive, confirmed by Western blot technique or DNA PCR, who were or were not on HAART. The exclusion criteria included children who were on medications with known cardiovascular effects, such as antiarrhythmic drugs, theophylline and adriamycin, children with pre-existing cardiac diseases, and children with other chronic diseases associated with demonstrable wasting or oedema.

Ninety patients who satisfied the study criteria were recruited after informed consent was obtained from their parents and other legal caregivers. Ethical approval was obtained from the ethics committee of the UNTH, Ituku-Ozalla, Enugu. Informed consent was obtained from parents or guardians of the children and older children, respectively.

All the sera from potential control subjects were screened for HIV infection using the Retrocheck® HIV testing kit (Nicholas Biotech, Texas, USA). Only those who tested negative were recruited for the study. The investigator administered a standard pre-test questionnaire to obtain biodata, demographic data and clinical history, including medication history, HIV and AIDS category based on CDC classification system, and type and duration of HAART. All subjects and controls also underwent a thorough physical examination.

The height and the weight were obtained using Hanson’s model H89 Orange® stadiometer and weighing scale respectively, according to standard procedures.^11^ Systolic and diastolic blood pressure measurements were taken on the right arm using an appropriately calibrated mercury sphygmomanometer with appropriate-sized cuff. The average of three readings was taken 10 minutes apart to represent the blood pressure estimate.

Full blood counts (FBC) were obtained on the I-STAT autoanalyser, and counter for haemoglobin concentration, leukocyte count and differentials, and erythrocyte sedimentation rate (ESR). CD4^+^ cell counts were obtained by auto-separation.

Echocardiography was done using the Hewlett-Packard SONO 2000 machine, which has a transducer with multifrequency in the range 5.5–12 MHz for children, a video recorder and a print-out processor. It has capabilities for M-mode, two-dimensional, pulsed wave and continuous-wave Doppler echocardiography. Echocardiography was performed on each child by two of the investigators and also interpreted to reduce intra-observer bias. These operators were blinded to the HIV and clinical status of the study subjects. For each examination, standard procedures and techniques were applied to windows.^13^ The younger subjects who were not cooperative in the presence of their caregiver or parents (usually those under two years) were pacified with toys or sedated with a mild short-acting sedative, chloral hydrate, as appropriate.

Echocardiographic measurements were taken in centimetres (cm) using the American Society of Echocardiography (ASE) guidelines for leading-edge methodology.^14^ The mean of three measurements was recorded and normative values for the echocardiographic measures, according to body surface area (BSA), were based on the ASE reference, as there were no local data available in this age group known to the authors at the time of the study.

Fractional shortening (FS) was calculated using the formula:

FS (%) = (LVEDd–LVESd) × 100/LVEDd

LVEDd = left ventricular end-diastolic dimension, LVESd = left ventricular end-systolic dimension. The normal range of FS is 28–41%, with a mean of 33 ± 5%.

Ejection fraction, EF (%) = stroke volume × 100/LVEDd

The normal range of EF is 45–90%, with a mean of 62 ± 10%.

Stroke volume (SV) = LVEDV–LVESV.

Left ventricular end-diastolic volume (LVEDV) = LVEDd^3^

Left ventricular end-systolic volume (LVESV) = LVESd^3^

Depressed LV systolic function is a fractional shortening of ≤ 28%, or ejection fraction of less than 40% with normal left ventricular dimensions.^14^

## Statistical analysis

Statistical analysis was done using the Statistical Package for Social Sciences (SPSS) version 18.0. Descriptive statistics for baseline demographic data are presented as both mean and standard deviation (SD) for continuous variables, or percentages for discrete variables. The non-parametric chi-squared (χ^2^) test was used to test comparable categorical variables, while one-way ANOVA was used for continuous variables. A value of *p* < 0.05 was considered statistically significant. Pearson’s correlation and multiple linear regression analysis were used to assess the relationship between left ventricular systolic dysfunction (LVSD) and the variables affecting it.

## Results

Table 1 shows the clinical and laboratory characteristics of the study participants. There were 90 children with HIV and AIDS, and 90 normal children were used as controls. Of the 90 with HIV and AIDS, 16 had clinical AIDS.

**Table 1:** Demographic and clinical characteristics of patients and controls

*Variable*	*HIV infection (n = 74)*	*AIDS (n = 16)*	*Control (n = 90)*	*F/χ^2^*	*p-value*
Gender					
Male	38	9	49	0.654	0.06
Female	36	7	41		
Mean age (years)	8.15 ± 3.08	7.9 ± 2.07	8.3 ± 3.04	0.14	0.87
Mean weight (kg)	14.43 ± 9.67	10.22 ± 6.07	22.4 ± 9.42	21.30	< 0.001
Mean height (cm)	108.1 ± 20.9	95.7 ± 15.3	114.7 ± 21.8	6.28	0.002
Mean BMI for age					
2–4 years (M)	18.3 ± 2	16.4 ± 2	22 ± 3.1	337.81	< 0.001
(F)	18.8 ± 1	16 ± 2	22.5 ± 2.1		
5–9 years (M)	16.7 ± 1	15.8 ± 1.2	23.2 ± 2.9	240.08	< 0.001
(F)	16.3 ± 1.2	15.5 ± 1.5	23.5 ± 2.4		
10–14 years (M)	17.5 ± 0.8	16.3 ± 2	21.5 ± 2	94.11	< 0.001
(F)	17.1 ± 0.9	16.3 ± 0.5	20.4 ± 3		
Mean RR/min	29 ± 5	32 ± 6	26 ± 5	13.12	< 0.001
Mean HR/min	103 ± 18	120 ± 20	92 ± 13	25.05	< 0.001
Mean SBP (mmHg)	89 ± 8	81 ± 12	85 ± 12	5.14	0.007
Mean DBP (mmHg)	52 ± 8	60 ± 7	54 ± 7	7.76	0.001
Mean Hb (g/dl)	9.8 ± 1.1	8.6 ± 0.7	11.6 ± 0.7	127.93	< 0.001
Mean WBC (cells/μl)	6813 ± 2056.3	4059 ± 1838.2	5059 ± 1838.2	23.02	< 0.001
Mean ESR (mm/1st h)	31 ± 10.6	67 ± 12.4	6.3 ± 2.4	486.40	< 0.001
Mean CD4^+^ (cell/mm^3^)	1486.6 ± 158.6	504.6 ± 300.3	1786.6 ± 1582.6	8.93	< 0.001
CD4^+^ (cell/mm^3^)					
≤ 1499, n (%)	6 (8.1)	15 (93)	30 (3.3)	5.6	0.01
≥ 1500, n (%)	68 (92)	1.1 (6.9)	87 (96)	4.54	0.05

BMI: body mass index, RR: respiratory rate, HR: heart rate, SBP: systolic blood pressure, DBP: diastolic blood pressure, Hb: haemoglobin, WBC: white blood cells, ESR: erythrocyte sedimentation rate.

There was no significant gender difference (χ^2^ = 0.654, *p* = 0.06) or difference in mean age between the groups. However there were significant differences in the mean weight, height, body mass index (BMI), respiratory rate (RR), heart rate (HR), systolic blood pressure (SBP), diastolic blood pressure (DBP), total white blood cell count, erythrocyte sedimentation rate (ESR) and CD4^+^ cell count between the controls, HIV and AIDS groups. The controls had higher weight, height, BMI, haemoglobin levels and CD4^+^ cell counts than the HIV and AIDS groups. The mean RR, HR and ESR were significantly higher in the HIV and AIDS groups than in the controls (*p* < 0.001). The AIDS group had severely depressed CD4^+^ cell counts compared to the other groups (χ^2^ = 5.6, *p* = 0.01).

Table 2 demonstrates the echocardiographic characteristics of the study participants with regard to systolic function of the heart. There was a significant difference in the mean left ventricular mass index (LVMI) of the HIV and AIDS groups compared with the controls. The LVMI was higher in the HIV and AIDS groups than in the controls. The mean FS and EF were significantly lower in the HIV and AIDS groups compared with the controls (*p* 0.001). The mean LVEDd and LVESd were significantly higher in the HIV and AIDS groups than in the controls. LVESd was highest in the AIDS group (Table 2). The prevalence of LVSD was highest in the AIDS group (81.2%), followed by the HIV-positive group (27%), and least (2.2%) in the controls. These differences were statistically significant (χ^2^ = 1.23, *p* = 0.03).

**Table 2:** Left ventricular echocardiography characteristics of the study participants

*Variable*	*HIV infection (n = 74)*	*AIDS (n = 16)*	*Control (n = 90)*	*F*	*p-value*
Mean LVMI (g/m^2^)	90.4 ± 25.3	89.4 ± 25.1	74.5 ± 23.2	9.47	< 0.001
Mean % FS	35.3 ± 10.5	31.6 ± 9.5	39 ± 5.2	7.75	0.001
Mean % EF	53.3 ± 15.7	45.3 ± 12.7	68.1 ± 12.4	39.922	< 0.001
Mean LVEDd (cm)	6.8 ± 0.6	6 ± 0.6	3.8 ± 0.7	441.89	< 0.001
Mean LVESd (cm)	2.7 ± 0.2	3.8 ± 0.4	2.2 ± 0.2	375.62	< 0.001
Prevalence of LVSD, n (%)	20 (27)	13 (81.2)	2 (2.2)	χ^2^ = 1.23	0.03

LVMI: left ventricular mass index, FS: fractional shortening, EF: ejection fraction, LVEDd: left ventricular end-diastolic dimension, LVESd: left ventricular endsystolic dimension, LVSD: left ventricular systolic dysfunction.

Table 3 shows the correlation of important determinants of cardiac systolic function in the HIV and AIDS groups. Age, duration of treatment, CD4^+^ cell count (in the HIV group) and pulse rate correlated positively with systolic dysfunction, while duration of treatment, diastolic blood pressure, and CD4^+^ cell count (in the AIDS group) demonstrated a negative correlation. Multiple linear regression analysis of factors that correlated significantly with LVSD revealed that age and CD4+ cell count were the best predictors of LVSD in our children who were HIV positive and in those with AIDS (*p* = 0.025 and 0.038, respectively) (Table 4).

**Table 3:** Pearson’s correlation of independent variables with LV systolic dysfunction in HIV carriers and AIDS groups

	*HIV carriers*		*AIDS*	
*Independent variable*	*Correlation coefficient (r)*	*p-value*	*Correlation coefficient (r)*	*p-value*
Age (years)	0.32	0.03*	0.22	0.01*
BMI for age	0.19	0.31	0.20	0.22
Duration of treatment (years)	–0.49	0.01*	–0.45	0.02*
SBP (mmHg)	–0.29	0.12	–0.30	0.45
DBP (mmHg)	–0.38	0.04*	–0.35	0.53
Haemoglobin conc (g/dl)	–0.20	0.30	–0.25	0.62
WBC (total)	–0.01	0.95	–0.05	0.12
ESR	–0.33	0.08	–0.35	0.24
CD4^+^ cell count	0.08	0.01*	–0.09	0.02*
Stage of disease	–0.05	0.32	–0.23	0.11
Pulse rate	0.13	0.04*	0.15	0.03*

**Table 4:** Stepwise multiple linear regressions of factors that correlated with LV systolic dysfunction in the subjects

	*Unstandardised coefficients*		*Standardised coefficients*			*95% CI for B*	
*Model*	*B*	*Std error*	*(r) Beta*	*t-value*	*p-value*	*B*	*Std error*
Constant	1.282	277		4.627	0.000	0.714	1.851
Age (years)	0.005	0.051	–0.212	1.170	0.025*	–0.015	0.004
CD4^+^ cell counts	0.034	0.016	0.396	2.186	0.038*	0.002	0.066

CI: confidence interval, dependent variable: LV systolic dysfunction, *Significant.

## Discussion

LVSD was more prevalent in the AIDS group (81.2%), than in the HIV group (27.0%) (*p* = 0.03). This is higher than the previous prevalences of 33.7% reported by Okoroma *et al.*^8^ in Lagos, Nigeria; 22% reported by Uwanuruochi^15^ in Enugu (in an adult population); and 29% reported by Lipshultz *et al.*^16^ in Boston. Other workers have reported wide-ranging figures for systolic dysfunction, such as the 6.5% prevalence noted by Cardoso *et al.*^9^ in Paris and 85.7% prevalence among adults reported by Longo-Mbenza1 in Kinshasa.

These observed differences in prevalence may have been due to the use of different criteria for the definition of cardiac abnormality, or methodological differences, including study design, sample size, patient selection method, focus on a single echocardiographic parameter and bias in patient selection in terms of inadequate matching for age and gender.^17^ However, these observed differences may also show that there is some racial or genetic predisposition to this detectable cardiac abnormality.^18^ In a multicentre, prospective cohort study conducted in the USA, the significance of a high prevalence of systolic dysfunction related to its association with mortality.^19^

The prevalence of cardiac dysfunction is high in African children with HIV/AIDS but this has not attracted much attention.^8^ This is partly because the clinical picture of HIV/ AIDS is still dominated by chronic diarrhoea from opportunistic infections, and severe malnutrition.^20^ Cardiac dysfunction is rarely diagnosed in HIV-infected children in our setting and standard care does not include echocardiography.^8^ Echocardiography is a non-invasive and valuable means of characterising cardiac abnormalities.

The mean weight and BMI in the AIDS group in our study was significantly lower than in the controls. This was expected as the loss of lean body mass, especially muscle protein, has been well documented in patients with HIV infection.^21^-^23^ Heart rate was significantly higher in the HIV and AIDS groups compared with the controls. Okeahialam *et al.* from Jos, Nigeria, noted this in 2000,^24^ and Coudray *et al.*^25^ reported similar findings in France. These findings may be as a result of ventricular dysfunction as well as autonomic dysfunction and the increased basal metabolic rate seen in HIV/AIDS patients.^24^

There were significant differences in the systolic and diastolic blood pressure of the HIV and AIDS groups in our study compared with the controls. Those with HIV or AIDS had higher blood pressure values than the controls. There are conflicting reports in the literature. Some workers found no differences in blood pressure,^26^-^28^ while others noted an increased frequency of systemic hypertension among patients with HIV/ AIDS.^13^,^24^ The compensatory mechanism of a normal or low blood pressure, seen in chronic malnutrition, which is prevalent in children with HIV/AIDS, may play a role.^29^ Haemoglobin level in the HIV/AIDS groups was significantly lower than in the controls. This was expected, due to chronic infection and malnutrition as a result of chronic diarrhoea.

The HIV group had significantly higher mean left ventricular end-diastolic dimensions than the controls. Fractional shortening and ejection fraction, on the other hand were significantly lower in the HIV and AIDS groups than in the controls, being lowest in the AIDS group. This was similar to the findings of Hecht *et al.*^30^ and Nzuobontane *et al.*^2^ They noted that end-diastolic dimensions were significantly higher in HIV-positive patients, while fractional shortening was significantly lower in AIDS subjects. This suggests that ventricular dilatation occurs earlier in the course of the disease than impaired contractility.

In identifying a possible link between certain variables and the presence of left ventricular systolic dysfunction, this study noted that BMI, blood pressure, except DBP (in the AIDS group), haemoglobin concentration, WBC, ESR and stage of the disease were not associated with the presence of systolic dysfunction (Table 3). Advanced stage of the disease, which is a known risk factor for cardiac involvement,^1^,^31^ was not significantly associated with the presence of LVSD in this study, even though the prevalence of LVSD was higher in the AIDS group. The reason for this was not obvious, however, it may be connected with the population studied, as racial or genetic differences had been noted.^18^ It is hoped that future studies will further investigate this finding.

Lower CD4^+^ count and younger age were significantly associated with the development of LVSD in the logistic regression model. This agrees with the report of Herskowitz *et al.*, who studied adults, and found a median CD4^+^ count of 30 cells/μl in HIV-infected patients with left ventricular dysfunction compared to a median count of 187 cells/ml in those without ventricular dysfunction.^32^ Lower CD4^+^ cell count is a marker of terminal disease associated with HIV cardiomyopathy, and younger children^20^ had been noted to have a rapid course of disease progression with end-organ effects.

Increased pulse rate was found in our study to be associated with LVSD, however, this was not noted by other investigators.^15^,^33^ This may not be unconnected with the population studied and the high prevalence of ventricular dysfunction observed in this study.

Multiple regression analysis showed that CD4^+^ cell count and age of the patients predicted the development of left ventricular systolic dysfunction, with CD4+ cell count being the best predictor (*r* = 0.396, CI = 0.002) (Table 4). This implies that significant decrease in CD4^+^ cell count was the highest risk factor for the development of LVSD in our subjects. This finding is at variance with Lipshultz *et al.*^16^ and Lobato *et al.*,^34^ who noted the presence of HIV encephalopathy as a predictor of LV dysfunction in HIV infection. This difference may have been due to the inclusion criteria, as only perinatal acquired HIV infection was included.

A limitation of the study is that the presence or absence of pre-existing cardiac abnormality prior to enrolment into the study was based on patients’ medical records or medical history. This did not completely exclude cardiac abnormality, as clinical evaluation alone is inadequate, as shown in the HIV-negative controls who had cardiac abnormalities.

## Conclusion

This study demonstrated a high prevalence of LVSD in children with HIV and AIDS, who apparently had no clinical evidence of heart failure. CD4^+^ cell count and age of the children were the best predictors of LVSD. The younger the age and the lower the CD4^+^ cell count, the higher the number of children with LVSD.

Since LVSD was asymptomatic in these children, it is recommended that HIV and AIDS children should undergo baseline and periodic evaluation using echocardiography. Cardiac care providers should be incorporated in the management of children with HIV/AIDS in our environment to implement appropriate preventative and therapeutic measures. This will maximise survival and improve the quality of life of these children.
